# The effect of prenatal counseling on breastfeeding self-efficacy and frequency of breastfeeding problems in mothers with previous unsuccessful breastfeeding: a randomized controlled clinical trial

**DOI:** 10.1186/s12905-020-00947-1

**Published:** 2020-05-05

**Authors:** Fahimeh Sehhatie Shafaei, Mojgan Mirghafourvand, Shiva Havizari

**Affiliations:** 1 Department of Midwifery, Faculty of Nursing and Midwifery, Tabriz University of Medical Sciences, Tabriz, Iran; 2 Social Determinants of Health Research Center, Tabriz University of Medical Sciences, Tabriz, Iran; 3 Student Research Center, Tabriz University of Medical Sciences, Tabriz, Iran

**Keywords:** Self-efficacy, Problems, Exclusive breastfeeding, Infant, Counseling

## Abstract

**Background:**

Breastfeeding is one of the most important interfering factors in infants’ health. Monitoring mothers’ performance and providing them with the feedback helps to increase their self-efficacy, interest in learning, and level of performance. The present research evaluates the effect of prenatal counseling on the breastfeeding self-efficacy and frequency of breastfeeding problems in mothers with previous unsuccessful breastfeeding.

**Methods:**

This randomized controlled clinical trial was conducted on 108 pregnant women with unsuccessful breastfeeding in Tabriz health centers during 2017–2018. The participants were randomly assigned to intervention and control groups. The intervention group had four prenatal counseling sessions and the controls only received routine care. Then, the mothers who gave birth to their children received a counseling session up to 4 months after the delivery. The Breastfeeding Self-Efficacy (BSES) questionnaire and the frequency of breast feeding problems checklist on the 15th day, and 2nd and 4th month were completed both by the intervention and control groups.

**Results:**

The mean (SD) of breastfeeding self-efficacy was 119.3 (10.5), 128.3 (8.3) and 133.8 (10.3) in the intervention group and 105.3 (16.1), 105.7 (19.7) and 109.4 (24.7) in the control group on the 15th day, 2nd and 4th month after the delivery, respectively. There was a significant difference in terms of breastfeeding self-efficacy between intervention and control group on the 15th day (*p* <  0.001), and 2nd (*p* <  0.001) and 4th (*p* <  0.001) month after the delivery. The frequency of breastfeeding problems on the 15th (*p* = 0.008), 2nd (*p* <  0.001) and 4th (*p* <  0.001) after the delivery was significantly different in most cases of the intervention group when compared to the controls.

**Conclusion:**

The results indicated that prenatal counseling can increase mothers’ breastfeeding self-efficacy and solves most breastfeeding problems during postpartum period.

****Trial registration**:**

IRCT20100109003027N19.

## Background

Exclusive breastfeeding has been defined as taking breast milk alone without consuming solids or other liquids except vitamins, minerals, and other medicines [1, 2], which are necessary for the infants in their the first 6 months of life [3–5]. Beside useful properties on infant’s well-being, breastfeeding reduces death rate caused by infectious diseases. Infants fed exclusively with breast milk are at a lower risk of gastrointestinal and allergic diseases [6].

Todays, the necessity of supporting and promoting breastfeeding is felt for infants’ health and growth, worldwide [7]. According to a report by World Health Organization (WHO), many countries in the Eastern-Mediterranean region, including Iran, have reported the high levels of onset and continuation of breastfeeding, annually. However, only approximately 42% of neonates are exclusively breast fed at their initial hours of life and approximately 41% of newborns have exclusive breastfeeding [8].

The experience of breastfeeding highly affects subsequent breastfeeding [9]. Due to the histories of failed breastfeeding and shorter breastfeeding periods in children because of previously unresolved breastfeeding problems and also low self-confidence, these mothers have an unpleasant experience in breastfeeding other children and this reduces their tendency to breastfeed [10].

Bandura has described self-efficacy as the capability of showing a behavior or doing something [11]. Breastfeeding self-efficacy is also associated with the mother’s perception of the adequacy of milk for her baby [12]. An study depicts that the level of self-efficacy in breastfeeding is related to a higher success rates in the initiation and continuation of breastfeeding [13], and makes mothers to think positive when facing breastfeeding problems and encounter with the challenges in more positive and efficient way [14].

Breastfeeding problems developed at postnatal phase create conditions that negatively affect breastfeeding period [15]. The problems often include thinking of inadequate milk, nipple scarring, congestion and insufficient evacuation of the breast, mastitis, breast abscess, and flattened nipples [16]. Breast pain caused by nipple scarring, mastitis, and mother’s concern about inadequate milk are the most frequent problems affecting almost 20–80% of women [17]. Breastfeeding is an action that needs to be learned [18]. The purpose of counseling is to help clients to better understand their surroundings and solve emotional and interpersonal problems [19]. A type of counseling is breastfeeding counseling in which theoretical and clinical aspects of breastfeeding are introduced together, and breastfeeding skills are practiced that include the observation and assessment of breastfeeding, helping mothers to hold their infants appropriately, and clinical management of common breast problems such as injured nipples, congestion, mastitis, and apparently insufficient milk [20].

Counseling on breastfeeding problems increases the level of exclusive breastfeeding [21]. Many studies have indicated the positive effects of counseling and breastfeeding interventions on the continuation of exclusive breastfeeding [9, 22, 23]; however, according to a conducted survey, no study was found on the effect of counseling on self-efficacy and breastfeeding problems in women with unsuccessful breastfeeding. Therefore, the present study was carried out to evaluate the effect of counseling on breastfeeding self-efficacy and the frequency of breastfeeding problems in women with unsuccessful breastfeeding.

## Methods

### Study design and participants

The present randomized controlled clinical trial was a part of a comprehensive study in which the effect of prenatal counseling on the breastfeeding practice of mothers with previous unsuccessful breastfeeding was evaluated and the breastfeeding self-efficacy and frequency of breastfeeding problems were measured as secondary outcomes. The results of primary outcomes have been published in another article [24]. This trial was conducted from November 2017 to May 2018 on 108 pregnant women who referred to the health centers in Tabriz, Iran. Inclusion criteria were the ability to read and write, residing in Tabriz, having previous unsuccessful breastfeeding, monogamy pregnancy, being able to constantly attend at counseling sessions, and being in the third trimester of their gestation. Exclusion criteria were fetal abnormalities, high-risk pregnancies, and breastfeeding contraindications.

The sample size was calculated based on the Kordi et al. study [25] and the “exclusive breastfeeding” variable. Forty nine participants were included in each group utilizing G-Power software and considering P_1_ = 40% (exclusive breastfeeding frequency in intervention group), P_2_ = 17.5% (exclusive breastfeeding frequency in control group), α = 0/05 and power = 80%. Finally, 54 participants were calculated to be the total sample size for each group, with a 10% of probable attrition rate.

### Study outcomes

The outcomes of this study were breastfeeding self-efficacy and frequency of breastfeeding problems in mothers with previous unsuccessful breastfeeding.

### Sampling and random allocation

The present study was approved by the committee of ethics of Tabriz University of Medical Sciences (IR.TBZMED.REC.1396.595) and was registered in Iranian Registry of Clinical Trials (IRCT20100109003027N19). Then, the samples were recruited utilizing available methods from all healthcare centers in Tabriz. After that, the researcher went to all healthcare centers and prepared a list of multipara pregnant women in their third trimester. The research aims were explained to eligible participants by phone calls and they were questioned on any previous unsuccessful breastfeeding experience. Then, they were evaluated in terms of inclusion and exclusion criteria in case of positive response to the former question and their readiness to contribute in the study. In case of eligibility, they would be invited to a briefing session. The study aims and method were explained and informed written consent was gained. Then, a socio-demographic questionnaire was completed. Participants were assigned into two groups, i.e. intervention recipient and control, using web www.random.org -based randomized block plan with block sizes of 4 and 6 and a 1:1 allocation ratio. Random allocation was done by an uninvolved person in sampling and data collection processes. Allocation concealment was done by writing the type of allocation on pieces of paper and placing them in consecutively numbered, opaque, sealed packets. Packets were opened in the order of participant entry in the study and they were allocated into either the counseling or control group.

### Intervention

In the intervention group, a set of breastfeeding counseling sessions in clusters of 5–7 participants, with each session for a period 60–90 min was begun. Four counseling sessions were held within a one-week time period and the control group only received routine care provided by healthcare center. The educational context in intervention group included the profits and combinations of breast milk, the psychological benefits of breastfeeding, breast structure and physiology, breastfeeding hormones, common reasons of breastfeeding discontinuation and failure, common breast conditions and disorders, maternal nourishment during breastfeeding, and breast pumping tips. Also, an instructive booklet was provided to the intervention group at the end of preliminary session. Phone call or, if necessary, face to face counseling was scheduled by the same consultant until the day 15 and up to the end of the 4th month postpartum in case of any trouble. A researcher-made checklist of frequency of breastfeeding problems and the standard Breastfeeding Self-Efficacy Scale (BSES) were completed for both groups on day 15 and months 2 and 4 postpartum.

### Data collection tool

In this study, self-efficacy was measured using self-efficacy measure (BSES) for breastfeeding. Breastfeeding self-efficacy includes 33 items and is scored on Likert Scale with the score 1 for “totally disagree” to score 5 for “totally agree”. The scores are 33 to165. Higher scores indicate higher levels of self-efficacy in breastfeeding. Bandura (1997) designed this tool, and Faux and Denis, for the first time, used it for breastfeeding [12]. Shahri et al. (2015) conducted the psychometric evaluation of the Persian version of this scale [26]. To determine the reliability BSES, test-retest analysis was carried out. The questionnaires were filled by 20 participants once, and the filling was repeated after 2 weeks, then the reliability was achieved by demonstrating in-line correlation coefficient and Cronbach alpha coefficient as 0.826 and 0.834, respectively, and then Confidence Interval (CI) was calculated to be 95%. In this study, the questionnaire was completed in three time intervals in the 15th day, 2nd and 4th month postpartum in both intervention and control groups. Breastfeeding problems were measured using a checklist of breastfeeding problems frequency designed by the present researchers (Additional file 1). Breastfeeding problems such as inadequate milk, baby’s refusal to take the breast, breast common conditions and complications, and baby’s complications were compared at three time intervals of 2nd and 4th months and 15 days postpartum in both intervention and control groups.

### Data analysis

SPSS software (version 24) was utilized for data analysis. The normal distribution of quantitative data was examined using the Kolmogorov–Smirnov test, indicating the normal distribution of all data. The socio-demographic features of both groups were compared using chi-square test, chi-square test for trend, independent t-test, and Fisher’s exact test. Analyses of the inter-group breastfeeding self-efficacy were conducted using chi-square test and repeated measure ANOVA test, the variables such as willingness to pregnancy, and mother/husband’s education were controlled. The inter-group frequency of breastfeeding problems were compared using chi-square and Fisher’s exact test. *p* <  0.05 was considered to be statistically significant.

## Results

### Participants’ characteristics

**Fig. 1:**
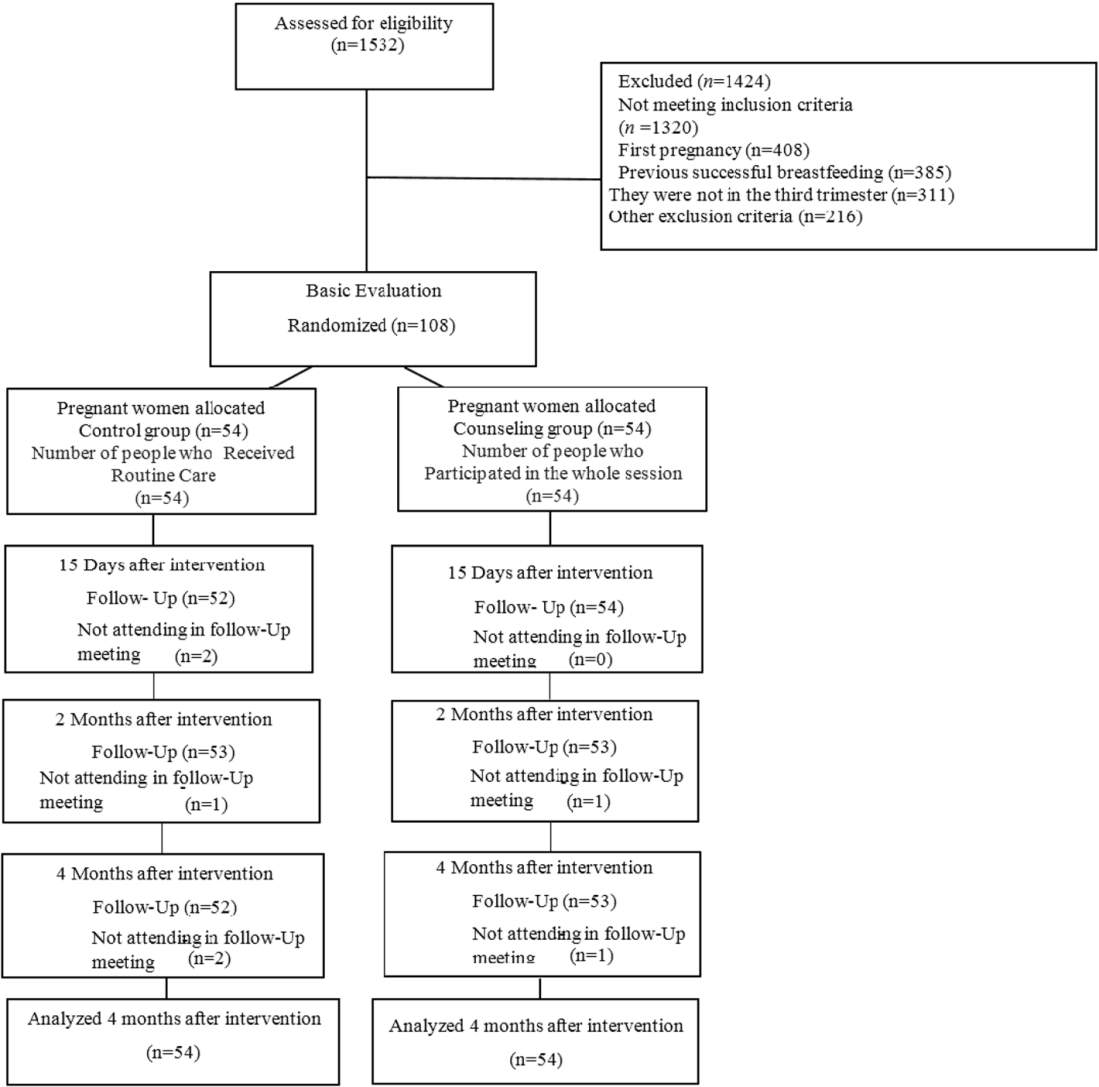
Trial Profile

The present randomized controlled clinical trial was conducted in November 2017 to May 2018.Out of 1532 screened pregnant women, 212 were eligible and 108 expressed their willingness to participate in the study and were randomly assigned to two groups of with 54 for each group. Fifteen days after the delivery, two individuals from control group, 2 months after delivery, one in the intervention and one in the control group, and 4 months after delivery, one from the intervention and two in the control group were excluded because they gave up to attend follow-up sessions. Finally, 101 individuals participated in the study. However, all subjects (108 people) were interviewed by phone call and filled out questionnaires (Fig. 1).

There was no significant difference between two groups in terms of socio-demographic features but in having tendency to pregnancy, and the level of mother and husband’s education the difference was significant. About one-third of mothers in the intervention group (37%) had diploma and in the controls (33.3%) had academic degree of education. About one-third and half of the husbands in the intervention (27.8%) and control groups (53.7%) had academic education, respectively. The majority of mothers in both groups (64.8% in intervention group and 81.5% in control group) inclined to be pregnant. Mothers’ mean age in both groups was about 30 years and the majority was housewives. About half of the participants in both groups (46.3% in intervention group and 50.0% in control group) mentioned insufficient breastfeeding as the breastfeeding problem in their former pregnancies (Table 1).

**Table 1:** Demographic characteristics of participants in the intervention (counselling) and control groups

Variables	counseling group(*n* = 54) n (%)^a^	Control group(*n* = 54) n (%)^a^	*P*-value
**Age**	32.3 (5.3) ^b^	30.2 (6.0) ^b^	0.056 ^c^
**Parity**			0.068^€^
1	28 (51.9)	39 (72.2)	
2	19 (35.2)	13 (24.1)	
3 ≤	7 (13.0)	2 (3.7)	
**Live child**			0.258^¥^
1	32 (59.3)	40 (74.1)	
2	20 (37/0)	13 (24.1)	
3	2 (3.7)	1 (1.9)	
**Willingness to pregnancy**			0.020^€^
Yes	35 (64.8)	44(81.5)	
No	13 (24.1)	10 (18.5)	
In future	6 (11.1)	0 (0.0)	
**Occupation**			0.153^¥^
Housekeeper	52 (96.3)	54 (100.0)	
Employed	2 (3.7)	0 (0.0)	
**Level of Education**			0.039^£^
Elementary	14 (25.9)	7 (13.0)	
Secondary	9 (16.7)	12 (22.2)	
High school	7 (13/0)	5 (9.3)	
Diploma	20 (37.0)	12 (22.2)	
University	4 (7.4)	18 (33.3)	
**spouse’s education**			0.011^£^
Illiterate	1 (1.9)	2 (3.7)	
Elementary	15 (27.8)	5 (9.3)	
Secondary	12 (22.2)	10 (18.5)	
High school	11 (20.4)	8 (14.8)	
University	15 (27.8)	29 (53.7)	
**Husband occupation**			0.419^¥^
Unemployed	1 (1.9)	1 (1.9)	
Manual worker	18 (33.3)	20 (37.0)	
Employee	11 (20.4)	11 (20.4)	
Shopkeeper	6 (11.1)	1 (1.9)	
Self-employment	18 (33.3)	21 (38.9)	
**Adequacy of monthly income**			0.376^£^
Quite enough	3 (5.6)	1 (1.9)	
Somewhat enough	43 (79.6)	43 (79.6)	
Not enough at all	8 (14.8)	10 (18.5)	
**Previous breastfeeding problem**			0.704^¥^
Inadequate milk	25 (46.3)	27 (50.0)	
Refusing to breastfeed	20 (37.0)	21 (38.9)	
Common problems and condition	9 (16.7)	6 (11.1)	

^a^ Number (Percent)

^b^Age variable was reported based on mean (Standard Deviation)

^c^ Independent T-test / ^€^ Fisher’s Exact test / ^¥^ Chi-square / ^£^ x^2^ for trend

**Fig. 2:**
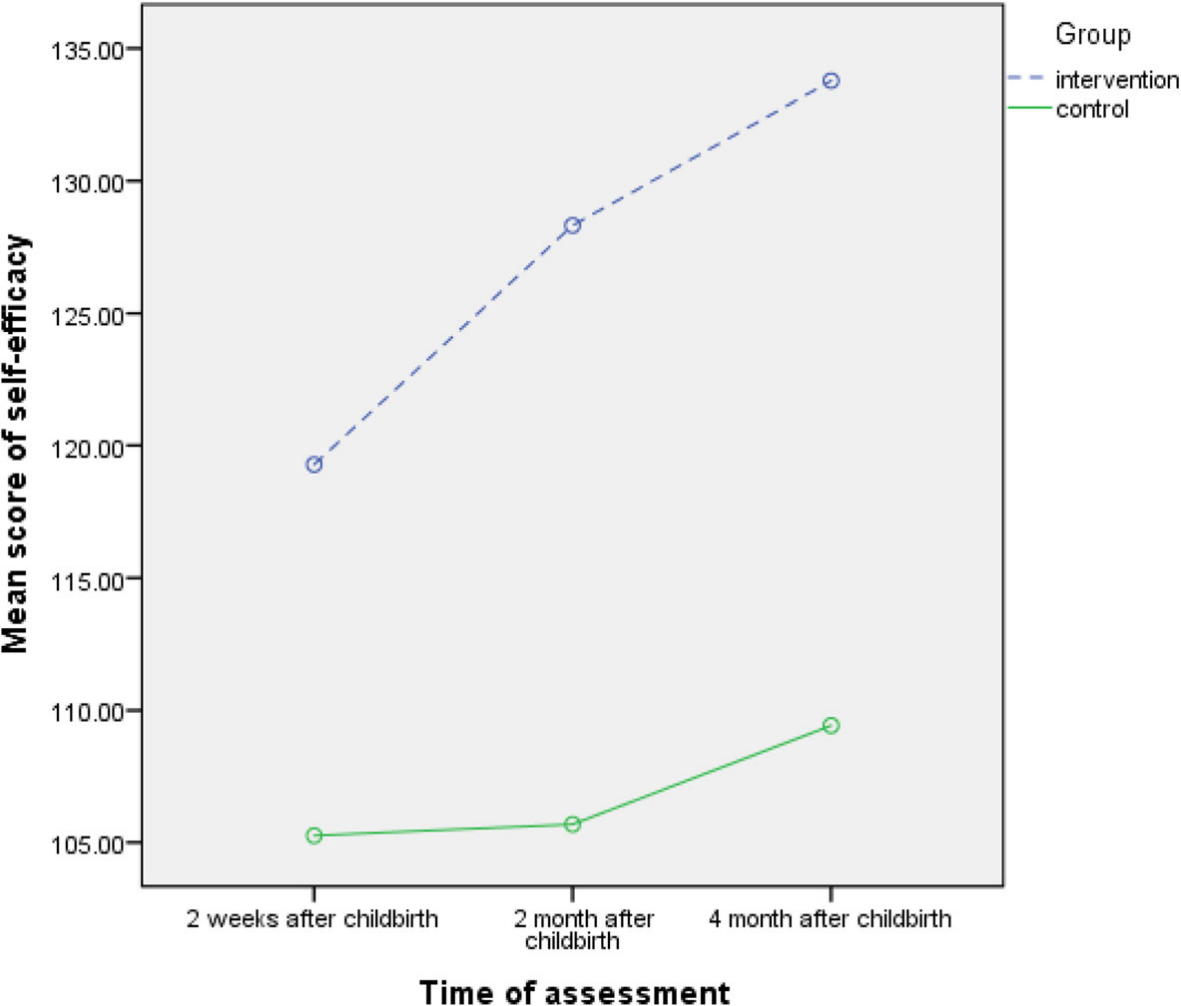
Comparison of the mean of self-efficacy score in mothers with Previous Unsuccessful Breastfeeding on days 15, 2 and 4 months postpartum in two groups of intervention (counseling) and control group

The mean (SD) score of self-efficacy on the 15th day after delivery in the intervention and control groups were respectively 119.3 (10.5) and 105.3 (16.1) indicating a significant difference between two groups (mean difference = 15.4; 95% confidence interval = 9.4 to 21.4; *p* < 0.001). Mean (SD) scores of self-efficacy in the 2nd and 4th months postpartum in intervention were respectively 128.3 (8.3) and 133.8 (10.3) and in control group they were respectively 105.7 (19.7) and 109.4 (24.7) indicating significant differences based on the ANCOVA test (mean difference = 24.6; 95% confidence interval = 17.7 to 31.5; *p* < 0.001) (mean difference = 25.3; 95% confidence interval = 16.7 to 34.0; *p* < 0.001). According to the results of repeated measure ANOVA and control of the variables of mother’s and husbands’ education and tendency to pregnancy, a significant difference was reported between the two groups in terms of mean scores of self-efficacy (adjusted mean difference = 21.8; 95% confidence interval = 15.3 to 28.2; p < 0.001). The interactive effect of time-group was also significant (*p* = 0.021) (Table 2) (Fig. 2).

**Table 2:** Comparison of the mean of self-efficacy score in mothers with Previous Unsuccessful Breastfeeding on days 15, 2 and 4 months postpartum in two groups of intervention (counseling) and control group

variables	Counseling group (*n* = 54) M (SD)	Control group (*n* = 54) M (SD)	Mean difference^a^ (CI =95%)^a^	P
Breastfeeding self-efficacy^b^ 15 days	119.3 (10.5)	105.3 (16.1)	15.4 (9.4 to 21.4)	< 0.001
Breastfeeding self-efficacy^b^ 2 months	128.3 (8.3)	105.7 (19.7)	24.6 (17.7 to 31.5)	< 0.001
Breastfeeding self-efficacy^b^ 4 months	133.8 (10.3)	109.4 (24.7)	25.3 (16.7 to 34)	< 0.001
Test results of repeated measure	Mean difference (CI =95%) P 21.8 (15.3 to 28.2) < 0.001		Time & Group Effect 0.001	Time Effect 0.021

^a^ANCOVA Test controlled such variables as willingness to pregnancy, level of mother’s education, and wife’s level of education

^b^ Breastfeeding self-efficacy has 33 items and is scored on Likert spectrum with score 1 for “totally disagree” to score 5 for “totally agree”. The distance between scores is from 33 to 165, and higher scores indicate a higher level of self-efficacy in breastfeeding

Comparison of breastfeeding frequency problems on the 15th day postpartum between the groups showed that the majority of mothers had fissure and breast pain, which were respectively 46.3 and 29.6% in intervention and control groups that showed a significant difference between the two groups (*p* = 0.008). In 27.8% mothers of intervention group and 22.2% of controls, baby’s sleeping prevented from enough breastfeeding (*p* = 0.075). Milk volume was adequate in 85.2% of the mothers in the intervention group and 38.9% in the control group that showed significant difference between the two groups (*p* < 0.001).

The comparison of breastfeeding frequency problems, 2 months postpartum, between two groups showed that most of the mothers in the intervention group (63.0%) had no problem at that time, and the impression of inadequate milk and infant’s crying due to hunger was observed only in 13% of the mothers of the intervention group, while in the control group, 46.3% of the mothers indicated this problem showing a significant difference between the two groups (*p* < 0.001).In the intervention group, 44.4% of the mothers, in 2 months postpartum, had no problem in performing breastfeeding. However, in the control group, 18.5% of the mothers indicated that they did not have regular and continuous breastfeeding showing a significant difference between the two groups (*p* < 0.001). 92.6% of mothers in the intervention group (50 cases) and 31.5% in the control group (17 cases) indicated milk adequacy, being indicative of increased milk adequacy when compared to 15 days after delivery (46 cases in the intervention group and 21 cases in control group). Here, the difference was also significant (*p* < 0.001).

Comparison of the breastfeeding frequency problems, 4 months postpartum indicated that most of the mothers in the intervention group (85.2%) had no problem at that time, and in the control group, more than one third of mothers (37.0%) still had the impression of inadequate milk and infant’s crying due to hunger problem, 20.4% had discontinuous and irregular breastfeeding and one fourth had the problem of milk inadequacy that indicated significant differences between the two groups (*p* < 0.001)(Table 3).

**Table 3:** Comparison of the frequency of breastfeeding problems on days 15, 2 and 4 months postpartum in two groups of intervention (counseling) and control group

Variable	Counseling group (*n* = 54) n (%)	Control group (*n* = 54) n (%)	P	Counseling group (*n* = 54) n (%)	Control group (*n* = 54) n(%)	P	Counseling group (*n* = 54) n (%)	Control group (*n* = 54) n(%)	P
	15 days			2 month			4 month		
**Breastfeeding problems**			0.008^✝^			< 0.001^✝^			< 0.001^✝^
Impression of inadequate milk and crying of the infant due to hunger	11 (20.4)	15 (27.8)		7 (13.0)	25 (46.3)		5 (9.3)	20 (37.0)	
Refusal to take breast	2 (3.7)	4 (7.4)		4 (7.4)	7 (13.0)		2 (3.7)	9 (16.7)	
Flattened and inverted nipples	3 (5.6)	9 (16.7)		1 (1.9)	1 (1.9)		–	–	
Breast Congestion	2 (3.7)	7 (13.0)		1 (1.9)	1 (1.9)		0 (0.0)	1 (1.9)	
Fissure and breast pain	25 (46.3)	16 (29.6)		1 (1.9)	2 (3.7)		–	–	
Mastitis	–	–		0 (0.0|)	2 (3.7)		–	–	
Fungal infection	0 (0.0)	1 (1.9)		6 (11.1)	5 (9.3)		1 (1.9)	2 (3.7)	
No problem	11 (20.4)	2 (3.7)		34 (63.0)	11 (20.4)		46 (85.2)	20 (37.0)	
**Breastfeeding performance problems**			0.075^✝^			< 0.001^✝^			< 0.001^✝^
Disrespect correct way of breastfeeding	9 (16.7)	5 (9.3)		1 (1.9)	2 (3.7)		–	–	
Infant sleeping and inadequate breastfeeding	15 (27.8)	12 (22.2)		10 (18.5)	6 (11.1)		2 (3.7)	2 (3.7)	
Irregular and discontinuous breastfeeding (in terms of time)	5 (9.3)	7 (13.0)		3 (5.6)	10 (18.5)		4 (7.4)	11 (20.4)	
discontinuous breastfeeding at night	4 (7.4)	7 (13.0)		1 (1.9)	4 (7.4)		0 (0.0)	3 (5.6)	
discontinuous breastfeeding From both breasts (in terms of lactation turn)	1 (1.9)	10 (18.5)		1 (1.9)	7 (13.0)		1 (1.9)	4 (7.4)	
Inadequate sucking due to infant’s low weight	3 (5.6)	2 (3.7)		0 (0.0)	2 (3.7)		1 (1.9)	4 (7.4)	
Inadequate sucking due to vomiting after breastfeeding	5 (9.3)	6 (11.1)		1 (1.9)	5 (9.3)		0 (0.0)	7 (13.0)	
Inadequate sucking due to other items	6 (11.1)	4 (7.4)		13 (24.0)	14 (25.9)		–	–	
No problem	6 (11.1)	1 (1.9)		24 (44.4)	4 (7.4)		46 (85.2)	18 (33.3)	
**Milk adequacy**			< 0.001^*^			< 0.001^*^			< 0.001^*^
Adequate	46 (85.2)	21 (38.9)		50 (92.6)	17 (31.5)		50 (92.6)	24 (44.4)	
Inadequate milk due to breastfeeding less than 8–12	3 (5.6)	16 (29.6)		2 (3.7)	12 (22.2)		3 (5.6)	13 (24.1)	
Inadequate milk due to inappropriate weight gain	3 (5.6)	10 (18.5)		1 (1.9)	9 (16.7)		1 (1.9)	7 (13.0)	
Inadequate milk due to the frequency of urine less than 6 times	1 (1.9)	1 (1.9)		–	–		–	–	
Inadequate milk due to the frequency of stool less than 2 times	1 (1.9)	6 (11.1)		1 (1.9)	16 (29.6)		0 (0.0)	10 (18.5)	

^*^Chi-square /^✝^Fisher’s Exact test

## Discussion

Breastfeeding counseling is effective on self-efficacy 4 months postpartum. Most of the studies in this field are consistent with the present study. A clinical trial in Canada was conducted by Noel-Weiss et al. (2006) on 110 primiparous women who participated in a workshop based on the theory of self-efficacy and breastfeeding principles. The results showed that the counseling affected self-efficacy and continuity of breastfeeding in primiparous mothers [27] with the only difference that the breastfeeding self-efficacy theory was not utilized in our study, and counseling was held in group meetings. The study in Wuhan,China (2014) indicated that interventions based on self-efficacy theory affected self-efficacy and short-term outcomes of breastfeeding in mothers [28]. Lingying et al. study (2016) indicated that a self-efficacy intervention on primiparous mothers’ breastfeeding behaviors was effective and helped mothers [29]. One study in Ahvaz, Iran (2018) was conducted on 120 primiparous women and the counseling group attended two counseling sessions before delivery and up to 8 weeks postpartum while the control group did not receive any intervention. The results showed that the mean score of breastfeeding self-efficacy in the intervention group was significantly higher when compared to the controls [30].This finding was in agreement with that of the present study. One study in Hong Kong (2016), also conducted on pregnant women who received an intervention in a workshop in the third trimester of pregnancy and received a phone call interviews 2 weeks postpartum, indicated that interventions only affected the amount of exclusive breastfeeding and did not affect breastfeeding self-efficacy [14], which did not confirm the results of our study. The reason for the lack of consistency can be the people cultural differences in this area and less intervention and follow-up sessions in the study.

Additionally, their findings confirmed that counseling on breastfeeding frequency problems did not affect breastfeeding 15 days postpartum, but it affected breastfeeding frequency problems 2 and 4 months postpartum. Feenstra et al. (2018) conducted a clinical trial on 1437 primiparous mothers and asked about breastfeeding experience shortly after the delivery. The findings indicated that 40% of the mothers had breastfeeding problems and most of the infant-related problems were associated with incorrect position of sucking the breast (40%) while most of the mother-related problems were related to fissures and nipple pains (38%). Factors associated to these problems included mothers’ first pregnancy, low self-efficacy, and mother’s knowledge on breastfeeding [31]. These results were consistent with that of the present study suggesting the prevalence of most common problems in the breast (fissure and breast pain) on the 15th day postpartum. In Kronborg et al. clinical trial (2009) they evaluated intervention group for breastfeeding methods at the first week postpartum and the follow up lasted for 6 months after delivery. The results indicated that half of the mothers in their first assessment after delivery used an incorrect breastfeeding method, the most important of which were incorrect position of breastfeeding (61%) and infant is not taking the breast (52%). The use of pacifiers was less related to the duration of exclusive breastfeeding. Their results suggested that monitoring breastfeeding methods during postpartum and not using pacifier could prevent occurrence of early and subsequent problems in breastfeeding [32]. This finding was also consistent with the results of our study. The only difference was that our study started from pregnancy and continued until delivery. In Flores et al. study on Bangladeshi women conducted on the counseling group, a counseling session was held in the last weeks of pregnancy, while the other group did not receive counseling. It was found that the amount of mastitis significantly decreased in the counseling group, and it affected subclinical mastitis and promoted the performance of breastfeeding [18]. In Henderson et al. clinical trial in South Australia on 160 primiparous mothers, the intervention group received structured breastfeeding interventions (on solving breastfeeding problems) and the control group received only routine training. After the delivery and within 24 h after birth, three and 6 weeks, and also 6 months postpartum, monitoring was conducted for exclusive breastfeeding, nipple pain, and scar problems. The results showed that the intervention group had less nipple pain in second and third days postpartum than the control group, but after the third day, no difference in pain scores was observed. There was no difference between the two groups in terms of nipple scars during the above-mentioned periods [33]. These findings are not consistent with that of our study on fissure and breast pain that showed a significant difference between two groups 15 days after delivery. The reason for this difference can be the holding of an educational session in the intervention group instead of several breastfeeding counseling sessions and immediate follow-ups after the delivery.

An study in Brazil (2006) was also conducted with 74 mothers in the intervention group and 137 mothers in the control group. The intervention group received 30-min postpartum counseling and was evaluated for breastfeeding problems during the first week and the first month after delivery. The results showed no statistically significant difference between the two groups in terms of nipple scars, breast mastitis, and congestion in the first week and 1 month after delivery suggesting that just an intervention cannot solve breastfeeding problems and also cannot increase the level of exclusive breastfeeding [34], these findings are not consistent with our study. In the present investigation, despite the presence of congestion, fissure, and breast pain after 15 days of delivery in both groups, the difference between the two groups was significant. The reason for this difference may include more counseling sessions and follow-up of mothers for breastfeeding problems up to 4 months after delivery in the present study.

## Strengths and limitations

Considering key features of clinical trials such as random allocation and allocation concealment, long-term follow-up of participants and relatively high sample size were the strengths of the present study.

The present study was only carried out in health centers and no direct supervision was done on breastfeeding process immediately after childbirth because mothers usually referred to health centers 15th postpartum that limited the present study. Therefore a similar study in which the mothers are supported to start breast-feeding immediately after the childbirth is recommended.

## Conclusion

The current findings suggest that prenatal counseling increases the frequency of breastfeeding problems and breastfeeding self-efficacy in mothers until 4 months postpartum. Counseling with mothers, particularly those with previously failed breastfeeding, in healthcare centers and by midwives and breastfeeding counselors, during exclusive breastfeeding period, can improve children’s health and well-being in the community.

## Supplementary information

**Additional file 1.** Breastfeeding questionnaire.

## Data Availability

Datasets used and analyzed during this study are available from the corresponding author on reasonable request.

## Abbreviations

IRCT: Iranian Registry of Clinical Trials
BSES: Breastfeeding Self-efficacy Questionnaire Scale
