# Supplementary material for: The effect of prenatal counseling on breastfeeding self-efficacy and frequency of breastfeeding problems in mothers with previous unsuccessful breastfeeding: a randomized controlled clinical trial
Source: BMC Womens Health. 2020 May 5;20:94. doi: 10.1186/s12905-020-00947-1 (PMC7201717; doi:10.1186/s12905-020-00947-1)
Supplement: Supplementary file 1 — Additional file 1. Breastfeeding questionnaire. [file 12905_2020_947_MOESM1_ESM.docx]

Appendix 1:

Questionnaires:

- Breastfeeding self-efficacy questionnaire

Reference : Dennis CL, Faux S. Development and psychometric testing of the Breastfeeding Self‐Efficacy Scale. Res Nurs health. 1999;22 (5):399-409.

- Breastfeeding problems frequency checklist

**Breastfeeding problems**

1. What problems do you have when breastfeed your infant?

| 1. Impression of inadequate milk and crying of the infant due to hunger |
| --- |
| 1. Refusal to take breast |
| 1. Flattened and inverted nipples |
| 1. Breast Congestion |
| 1. Fissure and breast pain |
| 1. Mastitis |
| 1. Fungal infection |
| 1. No problem   **Breastfeeding performance problems** |

2) Which one did you experience while breastfeeding?

| 1. Disrespect correct way of breastfeeding |
| --- |
| 1. Infant sleeping and inadequate breastfeeding |
| 1. Irregular and discontinuous breastfeeding (in terms of time) |
| 1. discontinuous breastfeeding at night |
| 1. discontinuous breastfeeding From both breasts (in terms of lactation turn) |
| 1. Inadequate sucking due to infant’s low weight |
| 1. Inadequate sucking due to vomiting after breastfeeding |
| 1. Inadequate sucking due to other items |
| 1. No problem |

**Milk adequacy**

3) Do you think you are breastfeeding well enough to meet your baby's needs?

a. yes

b. no

If not, which of the following indicates that you have not enough breastfeeding?

| 1. Inadequate milk due to breastfeeding less than 8-12 |
| --- |
| 1. Inadequate milk due to inappropriate weight gain |
| 1. Inadequate milk due to the frequency of urine less than 6 times |
| 1. Inadequate milk due to the frequency of stool less than 2 times |
